# Nano-Platelets as an Oxygen Regulator for Augmenting Starvation Therapy Against Hypoxic Tumor

**DOI:** 10.3389/fbioe.2020.571993

**Published:** 2020-09-04

**Authors:** Chunyu Huang, Chang Zhu, Jie Chen, Kaibin Huang, Fang Li, Shunkai Ding, Ligang Xia, Wei Jiang, Yang Li

**Affiliations:** ^1^Department of Gastrointestinal Surgery, Shenzhen People’s Hospital, The Second Clinical Medical College, Jinan University, The First Affiliated Hospital, Southern University of Science and Technology, Shenzhen, China; ^2^Key Laboratory of Artificial Micro- and Nano-Structures of Ministry of Education, School of Physics and Technology, Wuhan University, Wuhan, China; ^3^Center for Precision Medicine, The Second Affiliated Hospital of Zhengzhou University, Academy of Medical Sciences, Zhengzhou University, Zhengzhou, China

**Keywords:** metformin, glucose oxidase, platelet membrane, starvation therapy, respiratory suppression

## Abstract

The restriction of a tumor’s energy supply is proven to be an effective means of treatment. Glucose oxidase (GOx), an enzyme that catalyzes the conversion of glucose to glucolactone, producing oxygen and hydrogen peroxide in the process, has proved useful in this regard. However, hypoxia, which is implicated in tumor growth, has been found to mediate resistance to this type of tumor starvation. Here, we describe the design and testing of a platelet membrane mimetic, PMS, consisting of mesoporous silica nanoparticles (MSNs) loaded with metformin (MET) as an inner layer and platelet membranes (PM) as an outer layer that inhibits oxygen consumption by the tumor cells’ respiratory pathways and enhances the effectiveness of GOx. MET directly inhibits the activity of complex I in mitochondrial electron transport and is thus a potent inhibitor of cell respiration. PMS target tumor tissue effectively and, once internalized, MET can inhibit respiration. When oxygen is plentiful, GOx promotes glucose consumption, allowing amplification of its effects on tumor starvation. This combination of respiratory suppression by PMS and starvation therapy by GOx has been found to be effective in both targeting tumors and inhibiting their growth. It is hoped that this strategy will shed light on the development of next-generation tumor starvation treatments.

## Introduction

Increased proliferation places extensive demands on tumor cells’ metabolic processes compared to their normal somatic cell counterparts. Higher levels of nutrients are required for increased energy production and macromolecular synthesis (Hay, 2016; Icard et al., 2018). Tumor cells characteristically make use of aerobic glycolysis in addition to mitochondrial oxidative phosphorylation to meet their energy demands (Liberti and Locasale, 2016; Gao and Wei, 2017), Changes and rearrangements of tumor cells’ metabolic pathways occur to accommodate these higher energy requirements (Vander Heiden et al., 2009; Tennant et al., 2010). This is known as the Warburg effect (Gatenby and Gillies, 2004; Kim and Dang, 2006; Koppenol et al., 2011), This shift to aerobic glycolysis renders the cells more vulnerable to alterations in the cellular glucose supply. This is the principle behind the proposed cancer starvation therapy, which effectively starves the cells by depleting their glucose supply (Kim and Dang, 2006; Li et al., 2018). This approach has stimulated much interest (Fu et al., 2018; Yu et al., 2018; Xie et al., 2019) and the glucose oxidase GOx has attracted attention (Fu et al., 2019; Yang et al., 2019; Gao et al., 2020; Ren et al., 2020) for its role in the oxidation of glucose to gluconic acid, a reaction that can be harnessed to restrict the glucose supply to tumor cells (Jain, 2014; Gao et al., 2020).

However, a number of obstacles to GOx-based therapy have been encountered. These are a consequence of the complexity of the tumor microenvironment (TME). One particular obstacle is the occurrence of TME hypoxia which may adversely affect the rate of GOx catalysis of glucose and oxygen consumption. These effects aggravate the hypoxia and hence jeopardize the goal of the starvation therapy (Cheng et al., 2015; Jia et al., 2016; Song G. et al., 2016; Liu et al., 2018). It would be possible to enhance the anti-tumor effect of GOx by increasing the levels of oxygen within the tumor. This has been attempted by using hyperbaric oxygen inhalation, but the effectiveness of this approach has been complicated by poor microvascular systems at the tumor site which prevent oxygenation of the hyperbaric blood as well as resulting in potentially toxic levels of systemic oxygen. Several novel approaches such as using oxygen carriers such as hemoglobin or perfluorcarbon for oxygen delivery have been tested in animal models with promising results (Chen et al., 2015; Cheng et al., 2016; Li et al., 2017). The use of enzymes that produce oxygen within the tumor itself has also been proposed. These include naturally occurring enzymes such as catalase and nanozymes such as MnO_2_ and CuO (Chen et al., 2018; Sharma et al., 2018; Yu et al., 2019; Wang et al., 2020; Yang et al., 2020). However, all these approaches face major challenges, including low efficiency of oxygen production, premature oxygen leakage, and inadequate tumor microvessels. Furthermore, reoxygenation can also provide an impetus to the tumor, stimulating further proliferation.

Because of these complications, decreasing oxygen consumption is regarded as a better approach. Known *in vivo* respiratory depressants such as atovaquone (ATO) (Pernicova and Korbonits, 2014), nitric oxide (NO) (Morales and Morris, 2015), dichloroacetic acid (Song X. et al., 2016) and metformin (MET) (Ma et al., 2020) can inhibit tumor oxygen consumption, resulting in increased sensitivity of the cells to hypoxia-resistant treatments. Importantly, compared with other respiratory depressants, the FDA-approved metformin, as a commonly used drug in treating type II diabetes mellitus, has been approved by the FDA owing to its hydrophilicty and extremely low levels of toxic side effects. It is also reported that MET could directly inhibit the activity of NADH dehydrogenase (also called “complex I”) in the mitochondrial electron transport chain, thus producing a potent inhibition of cellular respiration. Thus, MET may be applied in combination with GOx to enhance starvation therapy. To the best of our best knowledge, a GOx and respiration inhibitor-encapsulating nanostructure, designed for promoting glucose consumption and enhancing its tumor starvation efficacy, has not been reported.

While some studies have been done to utilize targeting and immune evasion capacity of platelet membrane and starvation therapy for tumor therapy, Pan developed a DOX-loaded platelet-inspired nanovehicles to enhance cell-specific targeting. Li studied a mCGP system to realize amplified synergistic therapeutic effects of starvation therapy and photodynamic therapy (Wang et al., 2020). But they only used platelet membrane targeting alone, or coordinated starvation therapy with photodynamic therapy, without further amplifying starvation therapy. Furthermore, a simple combination of different treatments does not achieve the best synergistic amplification. It is urgent to develop a starvation-enhancing therapy.

**SCHEME 1:**
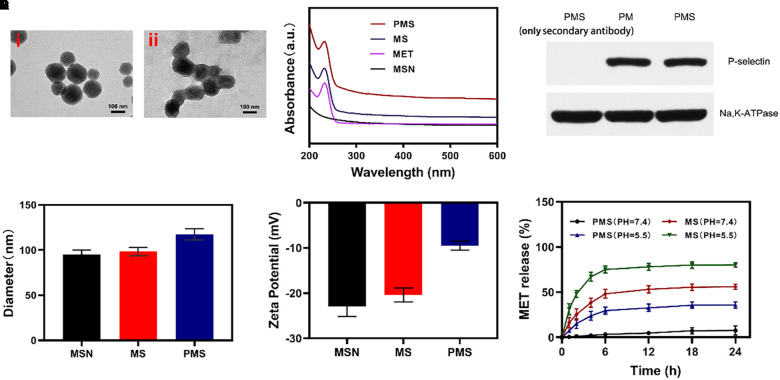
Schematic illustration of tumor-targeting PMS facilitate efficient respiratory suppression and starvation therapy.

Mesoporous silica nanoparticles have large surface areas (>1,000 m^2^/g), high pore volumes, and tunable pore sizes (2–20 nm) making them useful as drug delivery carriers (40). Based on the above literature research, in this work, we synthesized a platelet membrane biomimetic, PMS, functioning as a tumor cell respiratory regulator. This consisted of a mesoporous silica nanoparticle (MSN) supporting a MET-coated platelet membrane (PM) and has been shown to be effective in tumor targeting and drug delivery as well as having a long half-life in the circulation and convincing anti-cancer activities. PMS can deliver metformin to tumor tissues for the inhibition of cell respiration. In the tumor microenvironment, MET can be rapidly released from the PMS and accumulates at the tumor site to produce oxygen. Subsequent intraperitoneal injection of GOx can consume a large amount of glucose to achieve efficient starvation therapy (Scheme 1). We believe this is the first study to combine respiratory suppression with starvation therapy. The therapeutic effects demonstrated by both *in vitro* and *in vivo* experiments provide novel ideas for the clinical application of cancer starvation therapy.

## Materials and Methods

### Materials

Metformin (MET) was purchased from Aladdin Reagent (Shanghai, China). The MSNs used in this study were obtained from Shanghai Carboxyl Bio-pharmaceutical Technology Co., Ltd., (China). Phosphate buffer solution (PBS) was obtained from Thermo-Fisher (Waltham, MA, United States). 1,1′-dioctadecyl-3,3,3′,3′-tetramethylindocarbocyanine perchlorate (DiL), ATP assay kit, MTT Cell Proliferation and Cytotoxicity Assay Kit, 2-(4-Amidinophenyl)-6-indolecarbamidine dihydrochloride (DAPI) were purchased from Beyotime Company (China). All of the aqueous solutions were prepared using purified deionized (DI) water purified with a purification system (Direct-Q3, Millipore, United States). The other solvents used in this work were purchased from Sinopharm Chemical Reagent (China) and Aladdin-Reagent (China).

### Cell Culture

4T1 mouse breast cancer cell line cells were obtained from the Cell Bank of the Chinese Academy of Sciences and incubated in RPMI-1640 medium supplemented with 10% FBS in a humidified atmosphere at 37°C. Cell cultures under normoxic conditions (pO_2_: 21%) were maintained in a humidified incubator at 37°C in 5% CO_2_ and 95% air. Hypoxic conditions (pO_2_: 2%) were produced by placing cells in a hypoxic incubator (Moriguchi, Japan) in a mixture of 2% O_2_, 5% CO_2_, and 93% N_2_.

### Animal Models

Female BALB/c aged 4–5 week were purchased from Vital River Company (Beijing, China). 100 μl of 4T1 cell suspension (5 × 106 cells per ml) were subcutaneous injected into each mouse to establish the tumor models. All animal procedures were approved by the Institutional Animal Care and Use Committee of Zhengzhou University (Approval number: ZDYWYJY2019018).

### Preparation of MS and PMS

To prepare the MET loaded MSN (MS), the MET solution (10 mg dispersed in PBS) was dropwise added to 10 mL distilled water containing 10 mg MSN slowly, and stirred at room temperature overnight to reach the equilibrium state. The resulting solution was then centrifuged at 8000 rpm for 10 min, and washed with distilled water to remove the physically adsorbed MET.

Fresh mouse blood was centrifuged at 200 g to achieve Platelet-rich plasma (PRP) and then suspended in an equal volume of ACD solution (acid-citrate-dextrose). Next, the sample was centrifuged at 800 g to obtain the platelets. After lysis, the mixture of MS and platelet membrane was sonicated and then stirred for 12 h to. Thereafter, a polycarbonate membrane containing 200 nm pore size was used for extruding the mixture 10 times using an extruder.

### Characterization of the PMS Nanoparticles

The morphology structures of MSNs, MS, and PMS nanoparticles were observed by the TEM (JEOL-2100). UV–vis spectra of different samples were recorded by the UV–vis spectrophotometry Lambda 35 (Perkin-Elmer). Hydrodynamic diameter and zeta potential were detected by the dynamic light scattering (Nano-ZS ZEN3600). The column effluent was detected at 232 nm using an UV–vis detector and quantifi ed by comparing the peak areas with the standard curve. Drug loading efficiency (DLE) = (weight of loaded drug/weight of feeding drug) × 100% (Jia et al., 2016).

### Western Blotting for the Key Proteins in PM and PMS

The total cellular protein was extracted using a protein extraction kit (Dingguo, China). The extracted proteins were separated using SDS-PAGE electrophoresis. After electrophoresis, the gel was treated with Coomassie blue staining. Extraction of protein for western blot was performed as described above. The proteins were then transferred onto polyvinylidene fluoride (PVDF) membranes (Bio-Rad). This was followed by a blocking step for 1 h with 5% skim milk, and then the membrane was incubated with the primary antibody against P-selectin (Proteintech) overnight at 4°C using Na,K-ATPase as the control. Finally, the membrane was incubated with the secondary antibody for 1 h at room temperature.

### Drug Release Studies

MS and PMS were incubated with PBS (pH 7.4) or PBS (pH 5.5), respectively. At the given time points, the released MET was measured by UV–vis spectra.

### Evaluation of Cell Respiration

4T1 cells were seeded in six-well plate at a density of 1 × 10^6^ cells per well and incubated in 2 mL of RPMI-1640 medium for 24 h followed by the replacement of the culture medium with 3 mL of culture medium containing MS or PMS at a relative MET concentration of 20 μg/mL and incubated for 10 h under 21% O_2_ condition. Next, the culture medium was sealed with liquid paraffin, and the O_2_ level of each chamber was measured over time with an oxygen electrode. The oxygen electrode was immersed in the culture medium for 10 min of stabilization followed by an addition of 3 mL of liquid paraffin to cover the culture medium. Thereafter, the oxygen content was recorded every 20 s.

### Cellular Internalization Experiment

For intracellular PMS observation, 4T1 cells were seeded in a confocal microscopy dish at the density of 1 × 10^5^ cells/well. Twenty-four hours later, the cells were incubated with Dil labeled PMS (MET concentration: 20 μg/mL). After incubation for 0.5 h and 2 h, the cells were washed with PBS, stained with DAPI (10 μg/mL) and observed by CLSM.

### *In vitro* Cytotoxicity Assays

4T1 cells were seeded into 96-well plates at a density of 5 × 10^3^ per well. After 24 h of incubation, the media of the 96-well plates were discarded. Subsequently, GOx, MS or PMS (equivalent MET concentration: 20 μg/mL) were dispersed into fresh DMEM and then inoculated into the 96-well plates and cocultured in the normoxic (21% O2) cell or hypoxic (2% O2) culture environments for 6 h. After 2h dispersion of MS and PMS, different concentrations of GOx (0, 1.25, 2.5, and 5 μg/mL) was dispersed.

Cell viability was quantified by the 3-(4,5-dimethylthiazol-2-yl)-2,5-diphenyltetrazolium bromide (MTT) assay and calculated based on the following formula: cell viability = (OD 570 nm of the sample/OD 570 nm of the control) × 100%. The cell viability of the control group was set as 100%.

### ATP Level Assessment *in vitro*

To estimate intracellular ATP level after different treatments, 4T1 cells were cultured in a 12-well plate (seeding density: 1 × 10^5^ cells per well) under normoxia or hypoxia condition for 8 h. Subsequently, the cells were treated by MS and PMS (equivalent MET concentration: 20 μg/mL) for 12 h. NIR laser irradiation was carried out for 10 min where applicable. After treatments, the cells were lysed and the supernatant was centrifugally collected. Finally, intracellular ATP level was measured by a standard ATP assay kit.

### *In vivo* Hypoxia Evaluation

Tumor-bearing mice were divided into three groups and intravenously injected with 200 μL of saline, MS, or PMS for each group at a relative MET concentration of 10 mg/kg. A total of 12 h later, mice were sacrificed, and tumors were obtained for HIF-1α staining.

#### *In vitro* Immune Evasion Study

RAW 264.7 were seeded in 12-well plates and cultured for 12 h. Different concentrations of MS and PMS (i.e., MSNs dose of 25, 50, and 100 μg/mL) were added the medium, and the cells grown without any particles were used as control. Then the cells were washed three times and then incubated for 4 h at 37°C, 5% CO_2_, and then washed with PBS three times. To quantify nanoparticle uptake, 1 mL aqua regia was added to the cells. The mixture was left at room temperature for 12 h, followed by annealing at 70°C for 6 h to remove the acids. The sample was then re-suspended with 1 mL DI water and the Si content in each sample was determined by using an inductively coupled plasma-atomic emission spectrometer (ICP-AES; iris Intrepid II XSP, Thermo Elemental, United States).

#### *In vitro* Cancer Targeting Study

At first, MS and PMS were incubated with 4T1 cells for 4 h at 37°C. The cells were then washed with PBS several times, fixed with PFA for 30 min at room temperature, stained with DAPI and then imaged by using a confocal laser scanning microscope (CLSM; IX81, Olympus, Japan). The nanoparticle uptake by 4T1 cells was also investigated following to the steps as described in the section of *In Vitro* Immune Evasion Study.

#### *In vivo* Pharmacokinetics, and Distribution Study

BALB/c mice (*n* = 3) received an intravenous (i.v.) injection of 100 μL PBS containing MS or PMS (with equivalent MET dose of 10 mg/kg). At various time points after the injection (i.e., 0.5, 1, 2, 4, 6, 12, and 24 h), 20 μL blood plasma was collected from the tail veins, dissolved in hydrofluoric acid and then centrifugation at 10000 rpm for 10 min. Finally the supernatants were collected and Si element concentration was quantitatively analyzed by ICP-MS.

All of the mice were euthanized, and then we collected their major organs 24 h after injection to determine the biological distribution of the particles. As mentioned above, the Si content was measured using ICP-MS. Samples with high Si content (e.g., livers, spleens, and lungs) were diluted to ensure measurement accuracy. The Si content in the major organs was expressed in the unit of the percentage of tissue-injected dose (% ID/g).

#### *In vivo* Antitumor Study

5 × 10^6^ 4T1 cells suspended in 100 μl PBS were subcutaneously injected into each mouse to establish the tumor models. When tumor size reached approximately 200 mm^3^, the mice were divided randomly into 4 groups (each group included five mice): (1) a control group (PBS injection); (2) GOx; (3) GOx + MS, and (4) GOx + PMS. The MET dose was 10 mg/kg in group 3 and 4. The GOx dose in groups 2–4 was 10 mg/kg. The X-ray was performed 6 h after intravenous injection. Mice body weight and tumor volume in all groups were monitored every 2 days. Since day 1, the MS and PMS were intravenously infused via tail vein every 2 days, the GOx was intraperitoneally injected into mice after 12 h injection of MS and PMS. A caliper was employed to measure the tumor length and tumor width and the tumor volume was calculated according to following formula. Tumor volume = tumor length × tumor width2/2. After 16 days treatment, all the mice were sacrificed. The tumor tissues were weighed, and fixed in 4% neutral buffered formalin, processed routinely into paraffin, and sectioned at 4 μm. Then the sections were stained with ki-67 and finally examined by using a fluorescence microscope (IX81, Olympus, Japan).

#### *In vivo* Toxicity

Healthy Balb/c mice were i.v., injected with GOx + MS and GOx + PMS (10 mg/kg MET, 10 mg/kg GOx *n* = 3) or PBS. At 15th day post the injection, the blood samples from these mice (≈1 mL) were collected for blood biochemistry analysis. The major organs including heart, liver, spleen, lung, and kidney were harvested, fixed in 4% of formalin, embedded in paraffin, sectioned into 4 μm slices, stained with hematoxylin and eosin (H&E), and observed by an optical microscope (BX51, Olympus, Japan).

#### Statistical Analysis

Data analyses were conducted using the GraphPad Prism 5.0 software. Statistical Significance was calculated by one-way ANOVA using the Tukey post-test. ^∗^*P* < 0.05, ^∗∗^*P* < 0.01, ^∗∗∗^*P* < 0.001.

## Results and Discussion

### C Haracterization of PMS NPs

**FIGURE 1:**
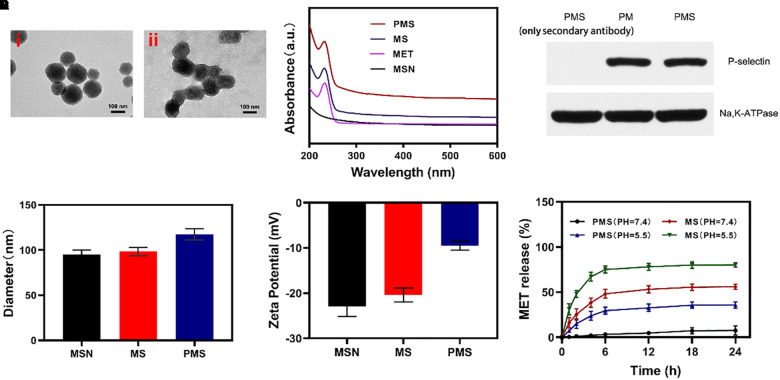
Characterization of PMS nanoparticles. **(A)** TEM image of (i) MSNs and (ii) PMS. **(B)** Absorbance spectrum of MSNs, MET, MS, and PMS. **(C)** The expressions of key protein P-selectin on PMS, with PM as a control. **(D)** Hydrodynamic diameter, and **(E)** Zeta potential of MSN, MS and PMS. **(F)** MET release profiles under different conditions.

Firstly, we successfully synthesized MSNs NPs, and loaded MET through mechanical mixing. The MET-loaded MSNs were shown by transmission electron microscopy (TEM) imaging to measure ∼100 nm in diameter with a gray 5 nm- thick membranous outer shell (Figure 1A). These characteristics were confirmed by UV–Vis spectrometry and dynamic light scattering (DLS). Successful encapsulation of NPs into membrane vesicles was demonstrated by the observation that, while the MSNs (96.8 ± 4.1 nm) and MS (98.4 ± 4.6 nm) were similar in size, the PMS (117.4 ± 5.8 nm) were slightly larger than the MS (Figure 1D). These findings were confirmed by the Zeta potential of the different particles (Figure 1E). The drug loading ratio was 40.3% for MET. Similar protein compositions were found for the PM-coated PMS NPs (Figure 1C), indicating that key proteins were unaffected by the preparation while characteristic PMS peaks near 232 nm were observed (Figure 1B). As shown in Supplementary Figures S3 and S4, no significant.

changes in the size or zeta potential of PMS following 3-day storage in PBS at 4°C, which indiacted that these nano partcles have longtime stability. Investigation of MET release from PMS NPs under different conditions (Figure 1F) showed that while MET was released from PMS NPs in acidic conditions, only small amounts were released at neutral pH. Compared to MS, MET release from PMS was less in both acidic and neutral conditions, indicating less leakage of the drug. The delay of MET release allows it to accumulate at the tumor site where it may act as an oxygen regulator to inhibit cell respiration. In addition, the action of GOx is likely to ensure sufficient oxygen to react with glucose to promote starvation.

### *In vitro* Tumor Cells Internalization

**FIGURE 2:**
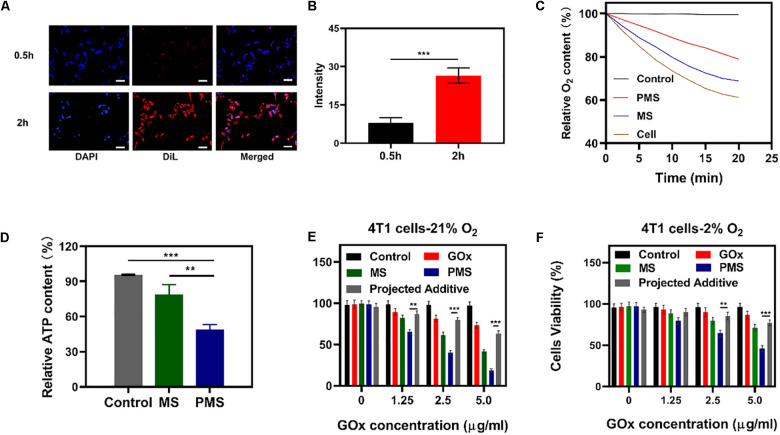
*In vitro* experiments. **(A,B)** Fluorescence images of 4T1 cells after treatment with PMS at different time point, respectively. Scale bars: 50 μm. **(C)** Relative oxygen content in the cell-containing medium under hypoxic condition (MET: 20 μg/mL). Blank control was detected in the cell-free medium. **(D)** Relative ATP content in the cells after 10 h co-culture with MS or PMS (MET: 20 μg/mL, *n* = 6). The survival of 4T1 cells with Control, GOx, MS, and PMS detected by MTT assay under **(E)** 21% O_2_ condition or **(E)** 2% O_2_ condition (*n* = 5). Statistical Significance was calculated by one-way ANOVA using the Tukey post-test. *P < 0.05, **P < 0.01, ***P < 0.001.

Internalization of the PMS nanoparticles was investigated using confocal microscopy. Images were analyzed with ImageJ software. The fluorescence images of 4T1 cells treated with PMS for varying times are shown in Figures 2A,B. It can be seen that the red PMS fluorescence increased over time, showing that PMS are readily taken up by tumor cells.

### Mitochondrial Respiratory Depression and ATP Depletion

Afterward, we assessed the impact of PMS on O_2_ as a function of incubation time. We then measured the oxygen consumption rate (OCR) by quantifying the concentration of oxygen dissolved in the medium. In cells cultured without PMS, the levels of oxygen dissolved in the culture medium diminished over time (Figure 2C), indicating consumption of oxygen. In the presence of MS and PMS, this oxygen consumption decreased significantly. This suggests that PMS reduces mitochondrial respiration. Tumors utilize more glucose than normal tissue and may use ATP generated by aerobic glycolysis to support their growth. We investigated ATP levels in 4T1 cells using an ATP assay kit. Figure 2D shows that the presence of PMS in the culture medium caused a significant reduction in the cells’ ATP levels, suggesting the effects of glucose depletion. These findings indicate that PMS is able to inhibit mitochondrial respiration, reducing both oxygen consumption and ATP production.

### Cell Viability *in vitro*

**FIGURE 3:**
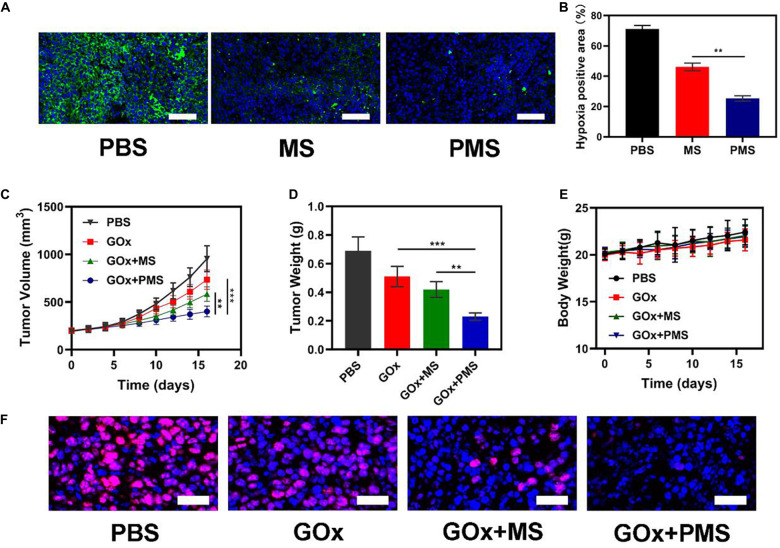
*In vivo* experiments. **(A)** HIF-1α staining of tumor tissues after different treatments. The scale bars are 50 μm. Blue indicated HIF-1α negative cells while green indicate HIF-1α positive cells. **(B)** Proportion of green cells in total (blue and green) cells. Data is shown as mean ± SD (*n* = 3). **(C)** tumor-volume change curves of 4T1 tumor bearing mice after various treatments. **(D)** Tumor weight change during therapy. **(E)** Body weight of 4T1 tumor-bearing mice recorded every two days after various treatments. **(F)** Representative Ki-67 (red) and DAPI (blue) stained tumor slice images of mice after various treatments. Scale bars: 50 nm. Statistical Significance was calculated by one-way ANOVA using the Tukey post-test. *P < 0.05, **P < 0.01, ***P < 0.001.

The anticancer effects of PMS on apoptosis were investigated using the cell apoptosis kit. In an environment with normal oxygen levels, significant cytoxicity was observed in the GOx group compared to the control group without GOx (Figure 3E). The level of apoptosis was highest in the PMS group which was, in turn, significantly higher than that seen in the MS group with a different GOx concentration. We attribute this to the synergistic effect of immune evasion of PM, GOx-mediated starvation treatment and MET-mediated respiratory depression. However, weak cytotoxicity was seen in the GOx groups in hypoxic environments (Figure 3F) indicating the necessity for sufficient oxygen for effective starvation therapy. It is noteworthy that cells in the PMS group underwent apoptosis in a hypoxic environment. Taken together, these results show that PMS is effective in inducing apoptosis in 4T1 cells. This indicates that the biomimetic regulatory system is effective in both normoxic and hypoxic environments.

### The Immune Evasion Function of PMS

Next, we explore the immune evasion function of MS and PMS nanoparticles were then incubated with RAW 264.7 cells, then we used inductively coupled plasma-atomic emission spectrometry to quantify the uptake. As shown in Supplementary Figure S5, PMS resulted in obvious lower uptakes, which indicating the ability of PMS to evade phagocytosis. Supplementary Figure S6 further demonstrate profound tumor targeting capacity of PMS.

### *In vivo* Pharmacokinetics and Biodistribution

We then conducted a pharmacokinetic experiment with PMS to examine the effects of the coated cancer membrane on blood retention of PMS. We intravenously injected SD mice with MS or PMS at a MET dose of 10mg/kg (Supplementary Figure S1). It was clear that, compared to MS, PMS showed significant blood retention. This indicates the efficacy of the cell membrane coating in prolonging the immune evasion ability of MSNs. We then investigated the biodistribution of MET in the MS and PMS groups (Supplementary Figure S2) using ICP-MS to measure the silicon content of tumor tissue and major organs after intravenous injection of MS or PMS. It was found that, while the silicon distribution in the heart and kidney was low, the tumor tissue and the liver showed a clear increase by 24 h post-injection. The MET distribution in the PMS group was higher than in the MS group, indicating the effective targeting ability of the coated cancer membrane.

### Anti-Hypoxia Capability of PMS *in vivo*

To monitor the anti-hypoxia capability of PMS *in vivo*, Hypoxia Inducible Factor-1α (HIF-1α) immunofluorescent staining was used to determine whether the hypoxia-related signaling pathways had been inhibited. It was found that HIF-1α levels were significantly higher in the PBS group compared to both the MS and PMS groups (PBS:0.713 ± 0.124; MS:0.313 ± 0.096; PMS:0.128 ± 0.045) (Figure 3B). The result demonstrates that PMS can significantly alleviate tumor hypoxia in the tumor microenvironment. Therefore, we postulate that a combination of GOx and MET and platelet membrane coating is likely to be highly effective as an anticancer treatment. The above results demonstrate that conventional starvation therapy might be suppressed due to hypoxia in TME and the introduction of MET could solve these problems.

### *In vivo* Antitumor Efficacy

The *in vivo* effects were further evaluated in nude mice with 4T1 tumor xenografts. When the tumor volume measured ≈200 mm^3^ (*n* = 5), the mice were divided into four groups: (1) PBS solution group, (2) GOx group (10 mg/kg). (3) GOx (10 mg/kg) + MS (10 mg/kg) group, and (4) GOx (10 mg/kg) + PMS (10 mg/kg) group. The volumes of the tumors were measured every two days with a digital caliper and the weights of the tumors were calculated. As shown in Figures 3C,D, the PBS group showed rapid tumor growth, while some inhibition of growth was noted in the GOx group. Meanwhile, the inhibitory effects of GOx + MS groups were slightly better than those in the PBS group. The GOx + PMS group showed significant suppression of both tumor volume and weight as the average weight of mice in the GOx + PMS groups was only 0.23 g, indicating that GOx + PMS had the strongest tumor inhibitory effect. No significant changes in the body weights of mice were observed in any of the groups (Figure 3E), indicating that the treatment strategy was effective with no obvious side effects. Furthermore, following treatment ki-67 staining (Figure 3F) was conducted, mice treated with GOx + PMS displayed the weakest cell proliferation signal. We attribute the superior anticancer efficacy of GOx-PMS to the combined effects of starvation therapy and suppression of mitochondrial respiration GOx is able to interfere with glucose metabolism by the conversion of glucose to gluconic acid under oxygen-sufficient conditions while the continuous inhibition of intracellular mitochondrial respiration through the introduction of MET in PM which can promote immune evasion and cancer targeting. All these results demonstrate that nano platelets acting as oxygen regulators may solve the problems of oxygen consumption in TME and thus combat the poor prognosis of tumors.

### Safety Evaluation

**FIGURE 4:**
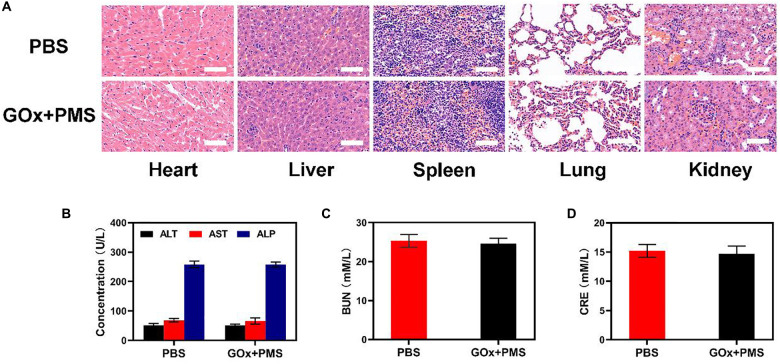
*In vivo* toxicity evaluation. **(A)** Histological data (H&E stained images) obtained from the major organs (heart, liver, spleen, lung, and kidney) of the different way treated mice 16 days postinjection under laser irradiation conditions (scale bars: 100 μm). **(B–D)** Blood biochemistry analysis (ALT, AST, ALP, BUN, and CRE) of healthy mice after intravenously injected with PBS or GOx + PMS for 14 days. *n* = 3, mean ± SD.

Sixteen days after the first injection, the mice were sacrificed and the liver, lungs, spleen, heart, and kidneys were examined for histological evidence of toxicity. No pathological changes or inflammation were observed in these organs (Figure 4A) indicating the potential biosafety of the treatment. In addition, serum levels of standard biochemical indices [alanine aminotransferase (ALT), aspartate transaminase (AST), alkaline phosphatase (ALP), blood urea nitrogen (BUN) and creatinine (CRE)] were normal indicating healthy kidney and liver functions (Figures 4B-D). Taken together, these results indicate the effective antitumor activity and biocompatibility of synergistic GOx and PMS, showing its potential as a cancer starvation therapy agent.

## Conclusion

In this study, a PMS system was successfully prepared to promote the efficiency of GOx as an antitumor agent. Importantly, GOx, as a glucose scavenger, could efficiently inhibit cancer growth under oxygen-sufficient conditions with no obvious side effects. FDA-approved metformin in PMS can depress mitochondrial respiration, leading to a decrease of endogenous oxygen consumption. Additionally, PCM is able to evade immune surveillance while targeting the cancer. The results indicate the efficacy and biosafety of the PMS-GOx combination as an anticancer treatment.

## Data Availability Statement

The raw data supporting the conclusions of this article will be made available by the authors, without undue reservation.

## Ethics Statement

The animal study was reviewed and approved by Institutional Animal Care and Use Committee of Zhengzhou University.

## Author Contributions

All authors listed have made a substantial, direct and intellectual contribution to the work, and approved it for publication.

## Conflict of Interest

The authors declare that the research was conducted in the absence of any commercial or financial relationships that could be construed as a potential conflict of interest.
