# Supplementary material for: Nano-Platelets as an Oxygen Regulator for Augmenting Starvation Therapy Against Hypoxic Tumor
Source: Front Bioeng Biotechnol. 2020 Sep 4;8:571993. doi: 10.3389/fbioe.2020.571993 (PMC7498539; doi:10.3389/fbioe.2020.571993)
Supplement: Supplementary file 1 [file Data_Sheet_1.docx]

**Nano-platelets as an oxygen regulator for augmenting starvation therapy against hypoxic tumor**

Chunyu Huang^1,3,#^, Chang Zhu^1#^, Jie Chen^1#^, Kaibin Huang^1^, Fang Li^1^, Shunkai Ding^1^, Ligang Xia^1*^, Wei Jiang^2*^ and Yang Li^1*^

^1^ *Department of Gastrointestinal Surgery, Shenzhen People’s Hospital （The Second Clinical Medical College，Jinan University；The First Affiliated Hospital, Southern University of Science and Technology）, Shenzhen 518020, Guangdong，China.*

*^2^ Center for Precision Medicine, the Second Affiliated Hospital of Zhengzhou University, Academy of Medical Sciences, Zhengzhou University, Zhengzhou 450001, China.*

*^3^ Key Laboratory of Artificial Micro- and Nano-Structures of Ministry of Education, School of Physics and Technology, Wuhan University, Wuhan, China*

^#^ C. H., C. Z. and J. C. contributed equally to this work**.**

^*^Corresponding authors: Email: ligangxiaszph@hotmail.com (L, Xia), [weijiang@zzu.edu.cn](mailto:weijiang@zzu.edu.cn) (W, Jiang); 1028168734@qq.com (Yang Li)





**Figure S1.** The pharmacokinetic behaviors of MS and PMS in mice after i.v. administration at MET dose of 10 mg/kg. Data were presented as mean ± SD (n = 3)





**Figure S2.** Quantitative analysis of Si biodistribution in tissues and tumors of tumor-bearing mice injected with MS and PMS at MET dose of 10 mg/kg.





**Figure S3.** DLS was used to measure the hydrodynamic diameter of MS and PMS.





**Figure S4.** The zeta potential of PMS suspended in PBS was assessed

after 1, 2, and 3 days

**

**

**Figure S5.** Nanoparticle uptake by RAW 264.7 cells at different incubated concentration (MSNs dose of 25, 50 and 100 μg/mL)

**

**

**Figure S6.** Nanoparticle uptake by 4T1 cells at different concentration. (MSNs dose of 25, 50 and 100 μg/mL)
